# Identification of Genetic Variants Causing Paediatric Cataract in Myanmar

**DOI:** 10.1111/cge.14755

**Published:** 2025-04-14

**Authors:** Johanna L. Jones, Daisy Boardman, Khine Nweni, Franoli Edo, Isabelly M. Barros de Lima, Pakdhipat Lertsinpakdee, Soe Hlaing, Robert Casson, Ashwin Mallipatna, Ye Win, Bennet McComish, Naing Lin, James Muecke, Andy Griffiths, Martin Holmes, Than Htun Aung, Kathryn P. Burdon

**Affiliations:** ^1^ Menzies Institute for Medical Research University of Tasmania Hobart Tasmania Australia; ^2^ Yangon Eye Hospital Yangon Myanmar; ^3^ Department of Ophthalmology Royal Adelaide Hospital and University of Adelaide Adelaide South Australia Australia; ^4^ Sight for All Adelaide South Australia Australia; ^5^ North Okkalapa General and Teaching Hospital Yangon Myanmar

**Keywords:** congenital cataract, genetic testing, rare diseases, splice assay, variant classification

## Abstract

Genetic testing for paediatric cataract detects a cause in 50%–70% of affected children but is as low as 20% in some reports. We screened 180 cataract‐related genes in 22 children (from 20 families) with paediatric cataract from Myanmar using whole‐exome sequencing. Pathogenic or likely pathogenic variants were identified in 45% (9/20) of probands in genes *MIP, COL2A1*, *NHS, GJA8, GJA3, CRYGC, CRYBB2, PAX6* and *SLC7A8.* Variants of uncertain significance likely to be important were identified in three children for a maximum diagnostic rate of 12/20 probands (60%) comparable to other reports. This is the first study to examine the genetics of paediatric cataract in Myanmar.

## Graphical abstract

Up to 60% of children with cataract in Myanmar have a causative variant in a known cataract gene. This is a similar rate to other populations screened to date, but highlights that there are more cataract genes left to identify.

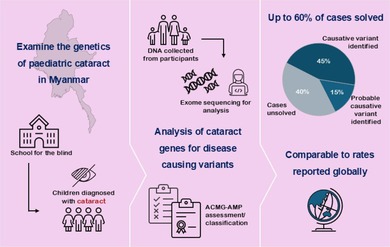

## 1 Introduction

Paediatric cataract is a heterogeneous disease characterised by an opacity of the ocular lens. It is a rare disease, with a prevalence of 0.6–10.86 per 10 000 births in Asia [1] and a well described genetic aetiology in a proportion of children [2]. Over 50 genes are linked to isolated paediatric cataract and many more when considering the variety of developmental syndromes that include cataract [2, 3]. Genetic testing typically provides a diagnosis for 50%–70% of affected children [4, 5, 6, 7]; however, this is not necessarily the case in populations of non‐European origin [8, 9]. Here we report findings from a screen of known cataract genes in 22 children from Myanmar diagnosed with paediatric cataract.

## 2 Methods

Children were included in this analysis if they participated in the parent study auditing causes of visual impairment and blindness among children in Myanmar [10], were reported to have paediatric cataract (± microphthalmia) and provided a saliva sample. Further details and exclusions are given in the Supporting Information. Whole‐exome sequencing, bioinformatics, variant filtering, variant classification, and a mini‐gene splice assay were carried out as described in Supporting Information.

## 3 Results

**FIGURE 1:**
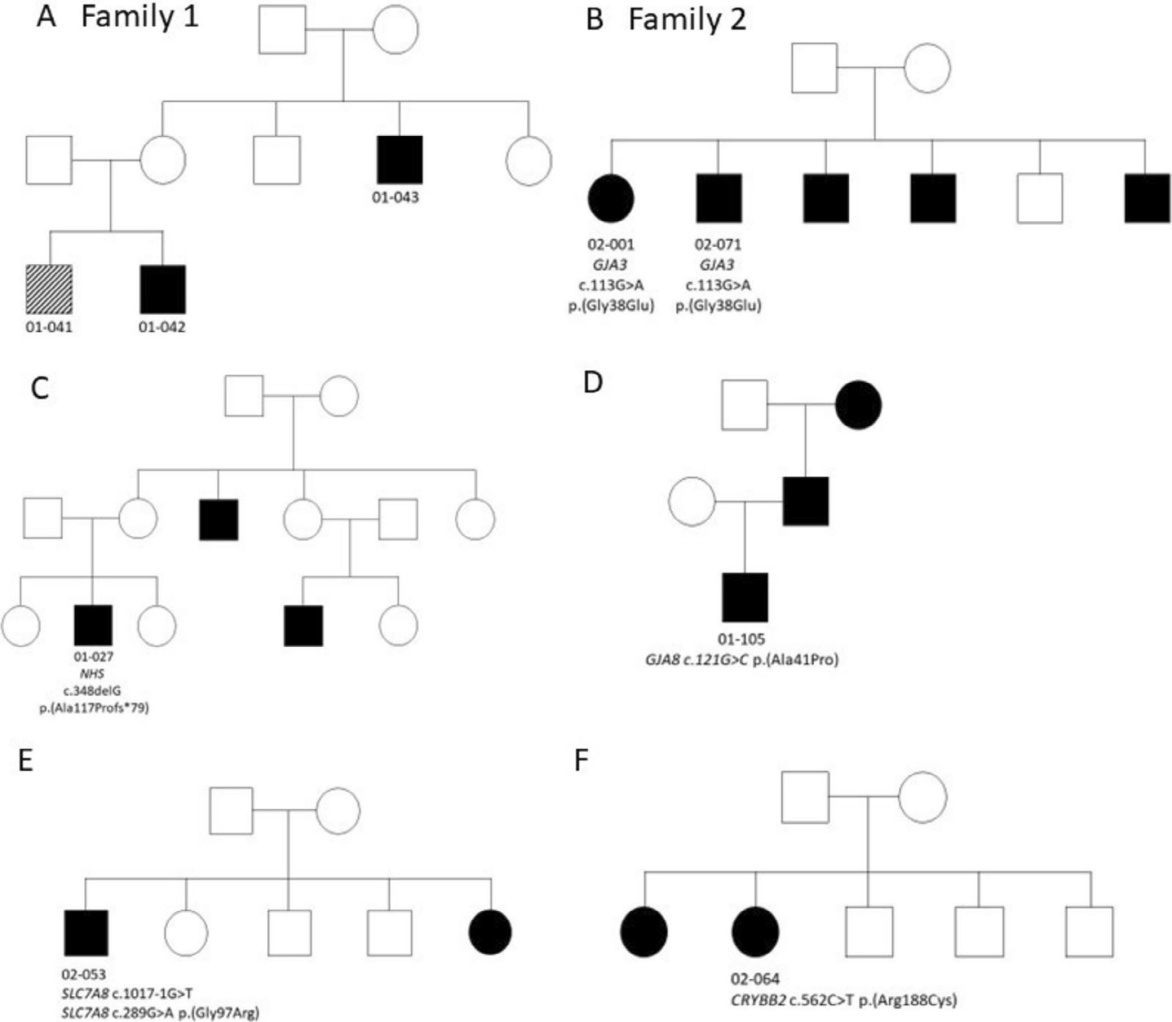
Pedigrees: Squares indicate males and circles indicate females. Individuals examined by study ophthalmologists are indicated by ID numbers. Filled symbols indicate affected with cataract and open symbols indicate unaffected. Hatched symbol indicates a non‐cataract ocular phenotype. (A) Family 1: No relevant variants detected. Individual 01‐041 had phthisis in right eye and opaque cornea with staphyloma in left eye and was not included in the exome sequencing analysis. (B) Family 2: VUS in *GJA3* gene (c.113G>A p.(Gly38Glu)). (C) Family of 01‐027 with *NHS* c.348delG p.(Ala117Profs*79) showing inheritance pattern consistent with X‐linkage. (D) Family of 01‐105 with VUS detected in *GJA8* showing an autosomal dominant inheritance pattern. (E) Family of 02‐053 with two variants in *SLC7A8*, consistent with autosomal recessive disease. (F) Family 02‐064 with *CRYBB2* c.562C>T p.(Arg188Cys) variant.

Of the 91 children recruited into this genetic sub‐study, 22 participants had paediatric cataract that was likely to have a genetic basis, including 18 singletons and two small families with multiple samples available (Table 1, Figure 1A,B). Thirteen participants reported a family history of childhood eye disease (Table 1). Microphthalmia was observed in 36% of cataract cases (8/22) and retinal or optic disc anomalies were observed in five children (Table 1).

**TABLE 1:** Clinical phenotype and most likely causative variant for each participant.

Sample ID		Family history	Phenotype	Sex	Age at exam	Age at onset	Surgery	Likely causative variant identified with ACMG classification
01‐001		None	Bilateral cataracts with amblyopia	M	15	0	Bilateral IOL at 9 years	*CRYBB2* NM_000496.3; c.565C>T, p.(Arg189Cys). VUS (homozygous)
01‐005		None	RE: Cataract, microphthalmia, corneal opacity LE: anophthalmia	F	14	0	Unknown	None
01‐008		Sister	Bilateral cataracts with amblyopia LE: Post Sx posterior capsule opacification	F	15	0	Bilateral IOL at 5 years	*MIP* NM_012064.4; c.638delG, p.(Glu213Valfs*46). Pathogenic
01‐013		None	High myopia with bilateral cataract RE: retinal detachment and retinal membrane. LE: retinal membrane	M	15	8	Bilateral membranectomy	*COL2A1* NM_001844.5; c.3137delC, p.(Pro1046Leufs*84). Pathogenic (Stickler Syndrome)
01‐014		None	Bilateral cataracts with post‐surgery corneal opacity	M	14	0	Cataract Sx at 4 years	None
01‐027		Cousin and uncle	Bilateral cataracts with microphthalmia and amblyopia	M	15	0	Cataract Sx at 12 years	*NHS* NM_001291867.2; c.348delG, p.(Ala117Profs*79). Likely Pathogenic
01‐032		None	Bilateral optic nerve atrophy RE: lamellar cataract	F	12	4	Unknown	None
01‐033		Sister	Bilateral cataracts with amblyopia	F	10	0	Bilateral IOL at 8 years	*GJA3* NM_021954.4; c.210C>G, p.(Phe70Leu). VUS
Family 1	01‐042	Uncle (01‐043)	RE: cataract with corneal scar LE: optic nerve hypoplasia	M	7	0	Unknown	None
	01‐043	Nephew (01‐042)	RE: corneal opacity LE: cataract	M	14	1	Unknown	None
01‐048		None	Bilateral cataracts with microphthalmia	M	11	0	Aphakic	*CRYBB2* NM_000496.3; c.464_469del, p.(Gln155_Tyr156del). VUS
01‐105		Father, grandmother	Bilateral cataracts	M	8	0	Unknown	*GJA8* NM_005267.5; c.121G>C, p.(Ala41Pro). Likely Pathogenic
Family 2	02‐001	Brother (02071)	Bilateral cataracts with amblyopia and corneal opacity	F	13	?	Unknown	*GJA3* NM_021954.4; c.113G>A, p.(Gly38Glu). Likely Pathogenic
	02‐071	Sister (02‐001)	Bilateral cataracts with amblyopia	M	13	2	Bilateral IOL at 7 years	
02‐013		None	RE: retrolental fibrous tissue LE: microphthalmia with complicated cataract	M	7	0	Unknown	None
02‐016		? younger brother	RE: microphthalmia with corneal opacity LE: microphthalmia with cataract	M	5	0	Unknown	None
02‐051		None	Bilateral cataracts with microphthalmia and amblyopia	F	15	0	Bilateral IOL at 8 years	*CRYGC* NM_020989.4; c.337C>T, p.(Gln113*). Likely Pathogenic
02‐053		Sister	Bilateral cataracts with amblyopia	M	15	0	Bilateral IOL	*SLC7A8* NM_012244.4; c.1017‐1G>T. Likely Pathogenic. c.289G>A, p.(Gly97Arg). VUS
02‐064		Sister	Bilateral cataracts with amblyopia	F	14	0	Bilateral IOL	*CRYBB2* NM_000496.3; c.562C>T, p.(Arg188Cys). Likely Pathogenic
02‐069		Sister	Bilateral cataracts with glaucoma in LE	F	15	0	Bilateral IOL at 3 and 11 years	*PAX6* NM_000280.6; c.802_806dup, p.(Leu271Lysfs*3). Likely Pathogenic
02‐072		Brother	Bilateral anterior polar cataracts with microphthalmia	M	12	1	Unknown	None
02‐083		None	RE: cataract with microphthalmia LE: morning glory disc anomaly	M	15	0	Unknown	None

*Note:* If applicable, related study participants are grouped together in a family. ‘None’ indicates no known family history of disease or no likely causative variants identified (see Table S2).

Abbreviations: F = female; IOL = intraocular lens implantation following cataract surgery; LE = left eye; M = male; RE = right eye; Sx = surgery; VUS = variant of uncertain significance.

A total of 45 high quality, rare, potentially deleterious variants were identified in the 180 genes analysed (Table S2). All children except one (01‐042) had variants meeting the filtering criteria. Variants classed as pathogenic or likely pathogenic were identified in 45% (9 of 20 probands) (Table 1, Table S2). Variants of uncertain significance that are highly likely to be the cause were detected in an additional 15% (3/20) of this cohort. No likely cause was identified in the remaining eight probands.

### 3.1 Pathogenic and Likely Pathogenic Variants

A previously reported pathogenic variant in *MIP* (c.638delG, p.(Glu213Valfs*46)) was detected in individual 01‐008 with bilateral cataract.

Individual 01‐013 has a single base deletion in *COL2A1* (c.3137delC, p.(Pro1046Leufs*84)). This individual has bilateral cataract, high myopia, bilateral retinal membranes and a retinal detachment in the right eye. This variant causes type 1 Stickler Syndrome (OMIM# 108300) [11, 12], a connective tissue disorder. The gene can also cause ocular Stickler Syndrome (OMIM# 609508) characterised by congenital vitreous abnormality consisting of a vestigial gel in the retrolental space, bounded by a highly folded membrane. The child having surgery for retinal membranes in both eyes is consistent with at least ocular Stickler Syndrome. There was no reported family history for this child, suggesting this variant likely occurred de novo.

A likely pathogenic frameshift c.348delG (p.(Ala117Profs*79)) variant in *NHS* was identified in male 01‐027 with bilateral cataracts, microphthalmia and amblyopia. His family history is consistent with X‐linked inheritance of the *NHS* gene (Figure 1C).

Individual 01‐105 carries *GJA8* c.121G>C p.(Ala41Pro) and reports an autosomal dominant inheritance pattern (Figure 1D) as is commonly reported for this gene [13]. Individuals 02‐001 and 02‐071 from Family 2 both carry *GJA3* c.113G>A p.(Gly38Glu) (Figure 1B).

In individual 02‐051, a stop‐gain variant in *CRYGC* (c.337C>T, p.(Gln113*)) was classified as likely pathogenic and would truncate the protein, disrupting a Greek key motif.

Two variants were detected in *SLC7A8* in individual 02‐053. The family history of this proband is consistent with autosomal recessive inheritance (Figure 1E), as previously described in the only other report of this gene with paediatric cataract [14]. A minigene splicing assay (Figure S1) showed that c.1017‐1G>T causes exon skipping resulting in a classification of likely pathogenic. The SpliceVault tool [15] indicates skipping of exon 8 in ~2% of events across the tissues represented in the database accessed by the tool. This is also consistent with the partial skipping of exon 8 in the wildtype construct of the mini‐gene assay (Figure S1). Skipping exon 8 is predicted to result in a frameshift that would undergo nonsense‐mediated decay. While this may be tolerated in small amounts, the completely null allele created by this variant would be consistent with a role in recessive disease. The second variant, c.289G>A p.(Gly97Arg), is a variant of uncertain significance. We cannot determine if the two variants are in trans, but it is very likely that they are the cause of cataract in this proband.

In individual 02‐064, a likely pathogenic variant, c.562C>T p.(Arg188Cys), was identified in the *CRYBB2* gene. This participant had bilateral cataracts with amblyopia and an affected sister who was unavailable for assessment (Figure 1F).

Individual 02‐069 carries a likely pathogenic frameshift variant in *PAX6*; c.802_806dup p.(Leu271Lysfs*3). This gene is well known to cause aniridia but has also been reported with isolated cataract phenotypes [16, 17] similar to those reported in this individual.

### 3.2 Variants of Uncertain Significance Likely to Be Important

Two VUS were identified in *CRYBB2*. Variant c.565C>T p.(Arg189Cys) is homozygous in individual 01‐001. Although autosomal dominant inheritance is more common, there are several reports of homozygous variants in *CRYBB2* causing recessive disease [18, 19]. An in‐frame 6 bp deletion c.464_469del p.(Gln155_Tyr156del) was observed in individual 01‐048, who has microphthalmia, a phenotype previously reported with *CRYBB2* cataract [20]. More evidence is needed for a formal likely pathogenic classification for both these variants; however, given the well documented role of this gene and this domain in paediatric cataract, it is likely they are involved.

A VUS of interest was also identified in individual 01‐033 in the *GJA3* gene, c.210C>G p.(Phe70Leu). This variant is in a hotspot for cataract‐causing variants in the first two transmembrane domains that form the pore of the connexon molecule [21].

## 4 Discussion

This is the first evaluation of childhood cataract genetics in Myanmar and provides important information, albeit with some limitations. As only minimal clinical and family history information was available, segregation could not be determined for most cases. Although we excluded children where intrauterine rubella or measles infections were suspected, some children may not have a genetic aetiology for their cataract. Some genes in this panel such as *FOXE3* and *MAF* have known challenges with coverage in exome sequencing, and causative variants in these small regions may have been missed. Nonetheless, this rate is broadly consistent with the 60%–70% observed in cohorts of European descent [4, 6] and higher than other South and East Asian cohorts reported [8]. The improved diagnostic rate is partly likely due to the expanded gene list used in this study detecting variants in COL2A1 and SLC7A8, which would have been missed using the panel used in our earlier study of Bhutan, Cambodia and Sri Lanka [8]. The genetic diagnosis rate may be further improved by inclusion of genes listed on panels for related disorders such as microphthalmia–anophthalmia–coloboma (MAC) syndrome; however, MAC genes linked to cataract were included.

We have identified the genetic cause of disease in 45% of the cohort (9/20) with pathogenic variants detected in *MIP and COL2A1* and likely pathogenic variants in *NHS, GJA8, GJA3, CRYGC, CRYBB2, PAX6* and *SLC7A8*. In addition, there are VUS of high interest in *CRYBB2* and *GJA3*. The genetic cause of cataract was identified in a minimum of 45% and a maximum of 60% (12/20) of the cohort, comparable to other populations, and we report only the second family worldwide with cataract likely to be caused by *SLC7A8*.

## Author Contributions

All authors contributed to the study conception and design. Data and specimen collection was performed by Khine Nweni, Franoli Edo, Soe Hlaing, Robert Casson, Ashwin Mallipatna, Ye Win, Naing Lin, James Muecke, Andy Griffiths, Martin Holmes, and Than Htun Aung. Sample processing, data generation, and experiments were performed by Johanna L. Jones, Daisy Boardman, Isabelly M. Barros de Lima, and Pakdhipat Lertsinpakdee. Data analysis was performed by Johanna L. Jones, Daisy Boardman, Bennet McComish, and Kathryn P. Burdon. The first draft of the manuscript was written by Johanna L. Jones, Daisy Boardman, and Kathryn P. Burdon, and all authors contributed to subsequent versions. All authors read and approved the final manuscript.

## Ethics Statement

Ethics approval was obtained from the Institutional Technical and Ethical Review Board, University of Public Health, Myanmar. Informed written consent was obtained from each participant, or their guardian, following a written and verbal explanation of the study given in the local language. The study was also approved by the Central Adelaide Local Health Network Research Ethics Committee (project 170173) and the Tasmanian Health and Medical Research Ethics Committee (project H0017509).

## Conflicts of Interest

The authors declare no conflicts of interest.

## Peer Review

The peer review history for this article is available at https://www.webofscience.com/api/gateway/wos/peer‐review/10.1111/cge.14755.

## Supporting information


Data S1.

Figure S1.

Table S1.
Table S2.

## Data Availability

Details of each variant are deposited in ClinVar (accession numbers SCV005081875 to SCV005081887). The genomic datasets generated during this study are not publicly available due to the requirements of the ethical review board to maintain privacy and anonymity of underage participants but may be available by request to the corresponding author with the agreement of the data custodians.
