# Supplementary material for: Identification of Genetic Variants Causing Paediatric Cataract in Myanmar
Source: Clin Genet. 2025 Apr 14;108(4):457–62. doi: 10.1111/cge.14755 (PMC12405064; doi:10.1111/cge.14755)
Supplement: Supplementary file 1 — Data S1. [file CGE-108-457-s002.docx]

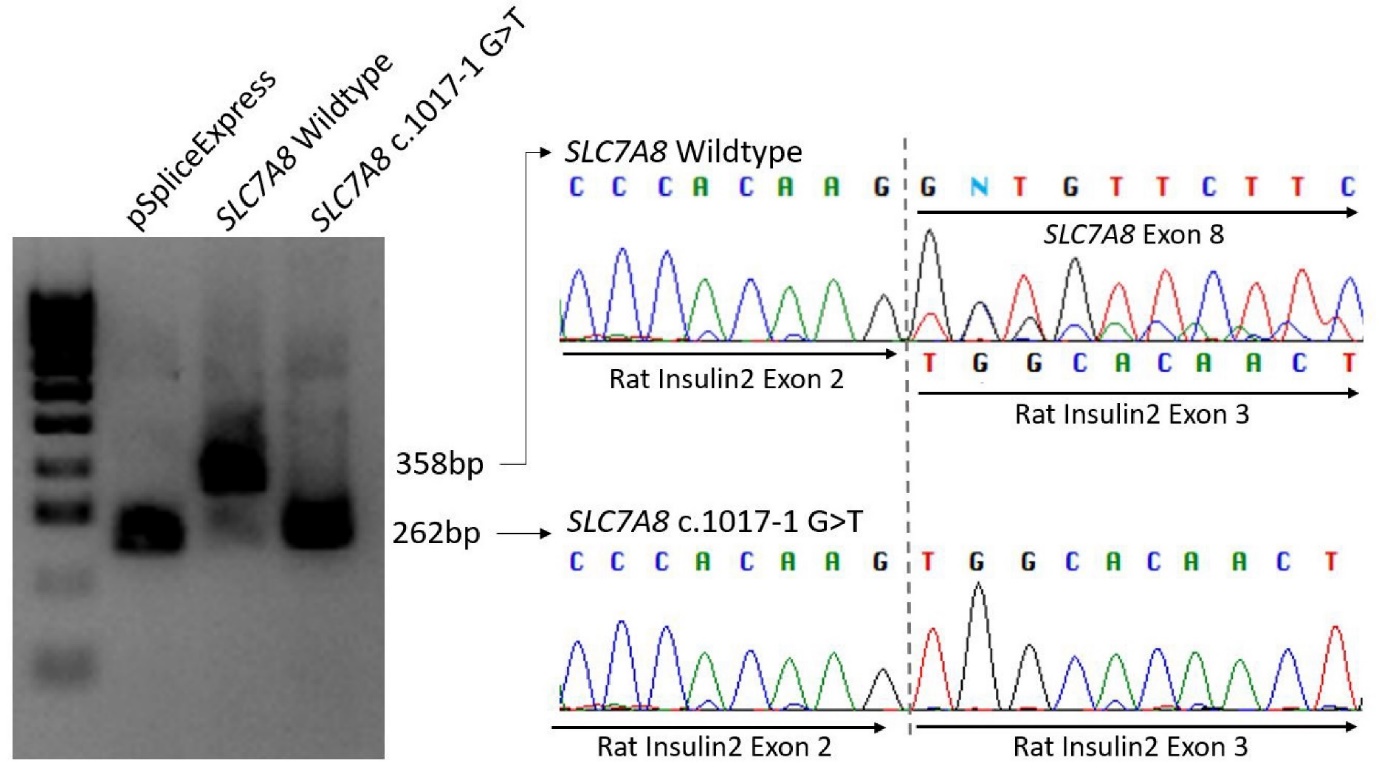


**Supplementary Figure 1**: Aberrant mRNA transcript splicing observed with the *SLC7A8* c.1017-1G>T variant in B-3 HLE cells. Sequencing chromatograms of the *SLC7A8* wildtype and c.1017-1G>T mutant constructs. *SLC7A8* c.1017-1G>T variant caused skipping of *SLC7A8* exon 8 producing a 262bp product and rat insulin exon-to-exon splicing consistent with the control pSpliceExpress plasmid. Wildtype *SLC7A8* plasmid produced a dominant RT-PCR product 358bp in size, as well as a smaller product. Sanger sequencing demonstrated that both *SLC7A8* exon retention (major band) and exon skipping (minor band) was occurring in the wildtype construct. Gel electrophoresis displayed with a 100bp ladder.
