# Supplementary material for: Identification of Genetic Variants Causing Paediatric Cataract in Myanmar
Source: Clin Genet. 2025 Apr 14;108(4):457–62. doi: 10.1111/cge.14755 (PMC12405064; doi:10.1111/cge.14755)
Supplement: Supplementary file 2 — Figure S1. [file CGE-108-457-s003.docx]

**Supplementary Methods**

##

## ***Study participants and DNA extraction***

Children under the age of 16 attending schools for the blind in Myanmar underwent ocular examinations by an ophthalmologist as part of a study to determine the causes of visual impairment and blindness among children in Myanmar ^1^. Demographic and family history data were obtained by interviewing children and their guardians. Children from two schools who were determined by the examining ophthalmologist likely to have a genetic cause for their cataract on the basis of the phenotype or a reported family history and who were willing to provide a saliva sample were also enrolled in a genetic sub-study, with the consent of their parent or guardian. Saliva samples were collected using the Oragene DNA saliva collection kit (DNA Genotek). Children were included in this analysis if they were reported to have paediatric cataract (± microphthalmia), had provided a saliva sample, and there was no indication of intrauterine rubella or measles infection or other non-genetic etiologies including trauma or corticosteroid use. Some participants had other features in addition to cataract noted. DNA was extracted from saliva samples using PrepIT-L2P reagent according to the manufacturer’s instructions (DNA Genotek).

## ***Whole-exome sequencing and variant classification methods***

Whole-exome sequencing was carried out using the Agilent SureSelect Human All Exon V7 library kit with 150bp paired-end sequencing on an Illumina Novaseq6000 platform at Macrogen, Inc. Burrows-Wheeler Aligner (BWA) ^2^ was used to align FASTQ files to the reference human genome, hg38, and variants were called using the Genome Analysis Toolkit (GATK) ^3,4^ implemented through the SAREK pipeline ^5^. Genes listed as ‘Green’ or ‘Amber’ status in PanelApp Australia (v0.355) or PanelApp Genomics England (v4.0) were included for screening as well as additional genes reported in the literature with a link to paediatric cataract (Supplementary Table 1).

Variants were annotated using ANNOVAR (version 2023Mar15) ^6^ and filtered based on the highest population minor allele frequency (MAF) in the Genome Aggregation Database (gnomAD, v4.0.0) ^7^. A minor allele frequency ≤ 0.00038 was used to capture rare variants based on a prevalence of paediatric cataract in Asia of 3.78 per 10,000 ^8^. Variants with CADD ^9^ (Combined Annotation Dependent Depletion, hg38 v1.7) PHRED scores ≥ 20, read depth >10 and genotype quality >20 were retained and visualised using the Integrative Genomics viewer (IGV) to confirm high quality sequencing with the variant allele present on reads in both directions. If one variant meeting filtering criteria for a recessive gene was identified in a proband, we manually looked for a second variant regardless of minor allele frequency. If both variants were more common than our filtering threshold, we will have missed the diagnosis. All were reviewed in gnomAD^7^, NCBI ClinVar, Cat-Map ^10^ (all accessed February 2024) and the general literature. UniProt ^11^ was used to determine a variant’s proximity to annotated protein regions.

Variants were classified according to the ACMG-AMP standards and guidelines for interpreting sequence variants ^12^ with specifications outlined in Methods Table 1. Points were awarded for each criterion as described in Tavtigian et al ^13^ and the sum of the point score was used to assign a classification (Pathogenic ≥10, Likely Pathogenic 6 to 9, VUS 0 to 5, Likely Benign -1 to -6 and Benign ≤-7).

**Methods Table 1**: Specifications applied to ACMG-AMP guidelines for the assessment of variants in paediatric cataract-related genes

| **ACMG-AMP rule** | **Specifications applied** |
| --- | --- |
| PVS1 (loss of function) | As per recommended decision tree ^14^ |
| PM2_supp  (population frequency) | Maximum population minor allele frequency (gnomad v4.0.0) <1.75x10^-7^, calculated with the Whiffin-Ware calculator. To calculate this threshold, genetic heterogeneity was set at 0.07 (the most commonly reported gene, *GJA3*, accounts for ~7% of cataract ^15^), allelic heterogeneity at 0.01 (no variant accounts for >1% of cataract), penetrance at 0.8 and prevalence at 1 in 2500 (reflecting the population prevalence of paediatric cataract in Asia ^8^) |
| PS4 (proband counting) | Supporting at ≥2 probands, Moderate at ≥ 6 probands, Strong at ≥ 15 probands ^16^, only if PM2_supp is applied |
| PP1 (segregations) | Supporting at ≥3 meioses, Moderate at ≥ 5 meioses, Strong at ≥ 7 meioses ^16^ |
| PP2 (constraint) | Missense variant constraint statistic (gnomAD v4.0.0) ≥3.1 |
| PP3/BP4 (computational predictions) | Strength thresholds ^17^, using REVEL for missense variants. Not applied if PVS1 or PM4 is applied) |
| PM1 (hotspot) | Amino acids 1-92 of *GJA3* (Moderate) or *GJA8* (Supporting) as reported previously ^18^ or in key domain indicated by UniProt containing other likely pathogenic or pathogenic variants. |
| PM5 (other missense variant) | Applied as supporting or moderate level depending on classification of the other variant as likely pathogenic or pathogenic, respectively. |
| All other rules | As per original recommendations ^12^ |

## ***Minigene splicing assay methods***

Predicted alteration of splicing of the SLC7A8 c.1017-1G>T variant was assessed using the pSpliceExpress splicing reporter system (Addgene plasmid #32485)^19^. Exonic and surrounding intronic sequence was amplified from DNA of heterozygous variant carrier individual 02-053 (Phusion® High-Fidelity PCR master mix with HF buffer, New England Biolabs), using Forward primer 5’-AAAAAGCAGGCTCCTTTGAGGATGTGAGGAATGC-3’ and Reverse primer 5’-AGAAAGCTGGGTCCAAAAGGATGGGAGAACGGA-3’. Subsequent nested PCR was performed with attB1 5’-GGGGACAAGTTTGTACAAAAAAGCAGGCT-3’ and attB2 5’-GGGGACCACTTTGTACAAGAAAGCTGGGT-3’ universal adapter primers ^19^ with PCR purification using Agencourt AMPure XP beads (Beckman Coulter). Gateway® BP Clonase™ II enzyme mix (Invitrogen) was used to recombine the PCR product into the pSpliceExpress plasmid immediately prior to transformation in One Shot® TOP10 Chemically Competent Escherichia coli (Invitrogen), per the manufacturer’s instructions. SLC7A8 c.1017-1G>T variant and wildtype colonies were identified using Isolate II Plasmid Mini Kit (Meridian Bioscience) and Sanger sequencing prior to obtaining larger quantities of desired colonies using Qiagen Plasmid Plus purification, all per manufacturer’s instructions.

The B-3 human lens epithelial (B-3 HLE) cell line (ATCC®CRL-11421™) was cultured in Gibco® Dulbecco’s Modified Eagle Medium (DMEM; Life Technologies) supplemented with penicillin/streptomycin solution (5000U/mL penicillin and 5mg/mL streptomycin (Sigma-Aldrich)) and 10% heat inactivated Gibco® fetal bovine serum (Life Technologies) at 37°C and 5% CO_2_. Lipofectamine™ 3000 reagent (Invitrogen™) was used to transfect B-3 HLE cells with two replicates of wildtype, variant and pSpliceExpress control plasmids using a 24-well plate layout. After 24 hours, cells were harvested and the RNeasy® Plus Mini Kit (Qiagen) was used to extract RNA prior to reverse transcription using oligo(dT)_20_ primers and the Invitrogen SuperScript® III First Strand Synthesis System (Thermo Fisher Scientific). RT-PCR with MyTaq HS mix (Meridian Bioscience) was performed using primers designed to bind to the constitutively expressed rat insulin exons present within the pSpliceExpress vector (RatIns2_Ex2_F 5’-CCTGCTCATCCTCTGGGAGC-3’ and RatIns2_Ex3_R 5’-ATGCTGGTGCAGCACTGAT-3’). Beta-actin primers were used assess cDNA quality and all products were visualised using agarose gel electrophoresis and Sanger sequencing using standard protocols.

**References for Supplementary Methods**

1. Huang S, Sun MT, Mallipatna A, et al. A survey of visual impairment and blindness in children attending eight schools for the blind in Myanmar: An update. *Indian J Ophthalmol*. Aug 2021;69(8):2034-2039. doi:10.4103/ijo.IJO_3534_20

2. Li H, Durbin R. Fast and accurate short read alignment with Burrows-Wheeler transform. *Bioinformatics*. Jul 15 2009;25(14):1754-60. doi:10.1093/bioinformatics/btp324

3. McKenna A, Hanna M, Banks E, et al. The Genome Analysis Toolkit: a MapReduce framework for analyzing next-generation DNA sequencing data. *Genome Res*. Sep 2010;20(9):1297-303. doi:10.1101/gr.107524.110

4. DePristo MA, Banks E, Poplin R, et al. A framework for variation discovery and genotyping using next-generation DNA sequencing data. *Nat Genet*. May 2011;43(5):491-8. doi:10.1038/ng.806

5. Garcia M, Juhos S, Larsson M, et al. Sarek: A portable workflow for whole-genome sequencing analysis of germline and somatic variants. *F1000Res*. 2020;9:63. doi:10.12688/f1000research.16665.2

6. Wang K, Li M, Hakonarson H. ANNOVAR: functional annotation of genetic variants from high-throughput sequencing data. *Nucleic Acids Res*. Sep 2010;38(16):e164. doi:10.1093/nar/gkq603

7. Karczewski KJ, Francioli LC, Tiao G, et al. The mutational constraint spectrum quantified from variation in 141,456 humans. *Nature*. May 2020;581(7809):434-443. doi:10.1038/s41586-020-2308-7

8. Tariq MA, Uddin QS, Ahmed B, Sheikh S, Ali U, Mohiuddin A. Prevalence of Pediatric Cataract in Asia: A Systematic Review and Meta-Analysis. *J Curr Ophthalmol*. Apr-Jun 2022;34(2):148-159. doi:10.4103/joco.joco_339_21

9. Rentzsch P, Schubach M, Shendure J, Kircher M. CADD-Splice-improving genome-wide variant effect prediction using deep learning-derived splice scores. *Genome Med*. Feb 22 2021;13(1):31. doi:10.1186/s13073-021-00835-9

10. Shiels A, Bennett TM, Hejtmancik JF. Cat-Map: putting cataract on the map. *Mol Vis*. 2010;16:2007-15.

11. Consortium U. UniProt: the universal protein knowledgebase in 2021. *Nucleic Acids Res*. Jan 8 2021;49(D1):D480-d489. doi:10.1093/nar/gkaa1100

12. Richards S, Aziz N, Bale S, et al. Standards and guidelines for the interpretation of sequence variants: a joint consensus recommendation of the American College of Medical Genetics and Genomics and the Association for Molecular Pathology. *Genet Med*. May 2015;17(5):405-24. doi:10.1038/gim.2015.30

13. Tavtigian SV, Harrison SM, Boucher KM, Biesecker LG. Fitting a naturally scaled point system to the ACMG/AMP variant classification guidelines. *Hum Mutat*. Oct 2020;41(10):1734-1737. doi:10.1002/humu.24088

14. Abou Tayoun AN, Pesaran T, DiStefano MT, et al. Recommendations for interpreting the loss of function PVS1 ACMG/AMP variant criterion. *Hum Mutat*. Nov 2018;39(11):1517-1524. doi:10.1002/humu.23626

15. Berry V, Georgiou M, Fujinami K, Quinlan R, Moore A, Michaelides M. Inherited cataracts: molecular genetics, clinical features, disease mechanisms and novel therapeutic approaches. *Br J Ophthalmol*. Oct 2020;104(10):1331-1337. doi:10.1136/bjophthalmol-2019-315282

16. Kelly MA, Caleshu C, Morales A, et al. Adaptation and validation of the ACMG/AMP variant classification framework for MYH7-associated inherited cardiomyopathies: recommendations by ClinGen's Inherited Cardiomyopathy Expert Panel. *Genet Med*. Mar 2018;20(3):351-359. doi:10.1038/gim.2017.218

17. Pejaver V, Byrne AB, Feng BJ, et al. Calibration of computational tools for missense variant pathogenicity classification and ClinGen recommendations for PP3/BP4 criteria. *Am J Hum Genet*. Dec 1 2022;109(12):2163-2177. doi:10.1016/j.ajhg.2022.10.013

18. Jones JL, Burdon KP. Evaluating gap junction variants for a role in pediatric cataract: an overview of the genetic landscape and clinical classification of variants in the GJA3 and GJA8 genes. *Expert Review of Ophthalmology*. 2023;18(1):71-95. doi:10.1080/17469899.2023.2160320

19. Kishore S, Khanna A, Stamm S. Rapid generation of splicing reporters with pSpliceExpress. *Gene*. Dec 31 2008;427(1-2):104-10. doi:10.1016/j.gene.2008.09.021
